# Artificial Intelligence-Enabled Electrocardiography for Preoperatively Detecting Atrial Fibrillation and Mortality Risk in Patients with Sinus Rhythm

**DOI:** 10.7150/ijms.123598

**Published:** 2026-01-14

**Authors:** Chiao-Chin Lee, Chin-Sheng Lin, Wen-Yu Lin, Chiao-Hsiang Chang, Wei-Ting Liu, Dung-Jang Tsai, Cheng-Chung Cheng, Jun-Ting Liou, Wei-Shiang Lin, Tien-Ping Tsao, Chien-Sung Tsai, Yung-Tsai Lee, Chin Lin

**Affiliations:** 1 Division of Cardiology, Department of Internal Medicine, Tri-Service General Hospital, National Defense Medical University, Taipei, Taiwan, R.O.C.; 2 Graduate Institute of Medical Sciences, College of Medicine, National Defense Medical University, Taipei, Taiwan, R.O.C.; 3 Military Digital Medical Center, Tri-Service General Hospital, National Defense Medical University, Taipei, Taiwan, R.O.C.; 4 Medical Technology Education Center, School of Medicine, College of Medicine, National Defense Medical University, Taipei, Taiwan, R.O.C.; 5 Department of Cardiology, Cheng Hsin Hospital, Taipei, Taiwan, R.O.C.; 6 Division of Cardiovascular Surgery, Department of Surgery, Tri-Service General Hospital, National Defense Medical University, Taipei, Taiwan, R.O.C.; 7 Division of Cardiovascular Surgery, Cheng Hsin Rehabilitation and Medical Center, Taipei, Taiwan, R.O.C.; 8 Graduate Institute of Life Sciences, College of Biomedical Sciences, National Defense Medical University, Taipei, Taiwan, R.O.C.

**Keywords:** atrial fibrillation, artificial intelligence, Mortality Risk

## Abstract

**Background:** Pre-existing atrial fibrillation (AF) and postoperative new-onset AF (NOAF) are independent perioperative risk factors associated with increased short-term mortality and adverse events. This study aimed to develop and validate an artificial intelligence (AI) model capable of detecting hidden AF, including both pre-existing AF and NOAF, from sinus rhythm electrocardiograms, to improve perioperative risks assessment.

**Methods:** We trained and validated an AI model to detect hidden AF. Subsequent analysis confirmed the prognostic relevance of both pre-existing AF and NOAF in patients receiving non-cardiac surgery. The AI model was applied to patients without known AF to evaluate its predictive capability for NOAF and to stratify short-term clinical outcomes.

**Results:** The AI model demonstrated an area under the receiver operating characteristic curve of 0.87 during the development phase for predicting AF. In an independent validation cohort, pre-existing AF and postoperative NOAF were significantly correlated with increased 30-day all-cause mortality. Patients without pre-existing AF who were classified as high-risk by the AI model had substantially higher 30-day all-cause mortality than their low-risk counterparts (HR 17.33, 95% CI 5.29-56.75). Furthermore, the model scores surpassed conventional clinical risk scores in predicting NOAF and 30-day all-cause mortality.

**Conclusions:** This AI-based approach facilitated the accurate identification of patients with elevated perioperative AF-related risk. It will facilitate focused interventions that may enhance clinical outcomes.

## Introduction

Atrial fibrillation (AF) is the most common and underdiagnosed arrhythmia, and it is associated with elevated risks of mortality, ischemic stroke, heart failure, and dementia.^1^-^3^ As a progressive condition, AF develops through a sequence of electrical, structural, and autonomic remodeling.^4^-^6^ Given that “AF begets AF,” early diagnosis has become essential in managing arrhythmia.^7^ Adequate treatment can slow disease progression, reduce complication risks, and minimize healthcare costs.^8^-^10^

Despite ongoing efforts, the underdiagnosis of AF is estimated at approximately 15%, with half of these patients having moderate-to-high risk of AF.^11^ Several studies showed that patients with new-onset AF (NOAF) have atrial substrate and autonomic remodeling similar to those with established AF.^5^, ^12^-^15^ Among patients undergoing surgery, preoperative pre-existing AF (pre-AF) and postoperative NOAF are independent risk factors that increase the risks of short-term mortality, ischemic stroke, myocardial infarction, and heart failure.^16^-^20^ However, undiagnosed pre-AF and the unpredictable nature of postoperative NOAF present significant challenges for anticipating or mitigating AF-related surgical risks.

Several risk schemes have been developed to accurately predict NOAF.^21^-^24^ All these scores require detailed clinical information and are limited to predicting long-term NOAF. In recent years, several deep learning models (DLM) have been developed to predict NOAF using a current sinus rhythm (SR) electrocardiogram (ECG), demonstrating impressive predictive capabilities.^25^-^28^ These studies have demonstrated that elevated AI-ECG predicted AF score are independently associated with higher risk of mortality and adverse cardiovascular outcomes, even in patients without a clinical diagnosis of AF at baseline.

Focusing on postoperative NOAF, existing model specifically have showed limited performance. A possible reasons is under diagnosis of postoperative NOAF, which makes the model training inaccurate. Therefore, we developed a DLM, AI-ECG, to detect patients with hidden AF, defined as those currently in SR who have pre-AF and those at risk of short-term NOAF in general population. In the study, we apply our AI-ECG to identify patients who were unaware of their AF-related perioperative risks and explore the short-term clinical outcomes.

## Methods

### Study Design

This retrospective cohort study was conducted at the Tri-Service General Hospital and its Tingjhou Branch in Taipei, Taiwan. We developed a DLM, referred to as the AI-ECG, to detect hidden AF using a 12-lead SR-ECG without the need for additional patient data. The development and validation processes for the AI-ECG are detailed in the supplementary materials. As shown in Supplementary Figure S2 and S3, the model demonstrated excellent performance in detecting hidden AF, pre-AF and NOAF, with AUCs 0.87-0.88, 0.87 and 0.89-0.91, respectively.

We assessed the AF-related perioperative risk in patients receiving non-cardiac surgery. Thereafter, we applied the AI-ECG to their preoperative sinus-rhythm ECGs to predict postoperative NOAF and stratify AF-related perioperative risk. This study was approved by the Institutional Review Board (IRB) of the Tri-Service General Hospital, National Defence Medical Centre (IRB no. C202105049).

### Study Population for Perioperative Risk Analysis

The study population comprised internal and external validation cohorts. Patients who did not undergo surgery, those who had cardiac surgery, and individuals without a 10-second, 12-lead SR ECG recorded within 3 days pre-surgery were excluded. Patients who were aware of AF-related perioperative risks and those receiving anticoagulant therapy, irrespective of the underlying reason, were also excluded. The study sample was stratified into three groups: patients with untreated pre-AF prior to surgery (pre-AF group), patients with NOAF within 30 days post-surgery (NOAF group), and the remaining patients categorized as the control group.

Risk of hidden AF and associated perioperative risk were assessed by the AI-ECG model using the preoperative 10-second, 12-lead SR ECG as input. The AI-ECG “high-risk” was defined by the cutoff point corresponding to a high positive predictive value (p > 0.994, Figure S3), while the “medium-risk” was defined by the cutoff point associated with high sensitivity (p > 0.047, Figure S2). Details of the cutoff selection are described in the Model Performance section of the Supplementary Materials. Clinical outcomes in the pre-AF, NOAF and control groups were evaluated according to the AI-ECG risk stratification.

### Clinical Outcomes and Variables

The primary outcome was 30-day all-cause mortality following surgery, while the secondary outcomes included new-onset ischemic stroke, acute myocardial infarction, and heart failure within the same 30-day period. Baseline characteristics, underlying diseases, and preoperative laboratory data were obtained from the electronic health records of all enrolled patients. The relevant preoperative data used for calculating the CHA2DS2-VASc score^29^ (congestive heart failure, hypertension, age, diabetes mellitus, stroke or transient ischemic stroke, vascular disease, and sex) and revised cardiac risk index (RCRI)^30^ (ischemic heart disease, congestive heart failure, cerebrovascular disease, insulin treatment, creatinine > 2 mg/dL, and elevated-risk surgery) were identified. Data necessary for calculating the C2HEST score and Taiwan AF score (TWAFS) were collected to facilitate a comparison between AI-ECG performance and current AF prediction scores using data specific to the Asian population.^21^, ^22^

### Statistical Analysis

For baseline characteristics, categorical variables are reported as numbers and percentages, while continuous variables are presented as means and standard deviations. Student's t-test or chi-squared test were used for comparisons, with p-values of < 0.05 deemed statistically significant. ROC curves and AUCs were employed to evaluate the performances of the AI-ECG model, C2HEST, and TWAFS scores. The Kaplan-Meier method was applied to manage censored data and calculate the cumulative incidence test, and the Cox proportional hazards model was used to estimate hazard ratios (HRs) with 95% confidence intervals (CIs).

Given the baseline characteristic differences among the three groups, logistic regression analysis was performed, incorporating variables such as surgery type, sex, age, CHA2DS2-VASc score, and RCRI to estimate the propensity score. The inverse probability weighting of propensity scores (IPWPS) approach was then applied to mitigate potential confounding bias while retaining the full sample.

### Code Availability

The code may be provided by the authors upon reasonable request, subject to permission and approval from the corresponding organizations and institutional review boards.

## Results

### Study Population and Baseline Characteristics

**Figure 1:**
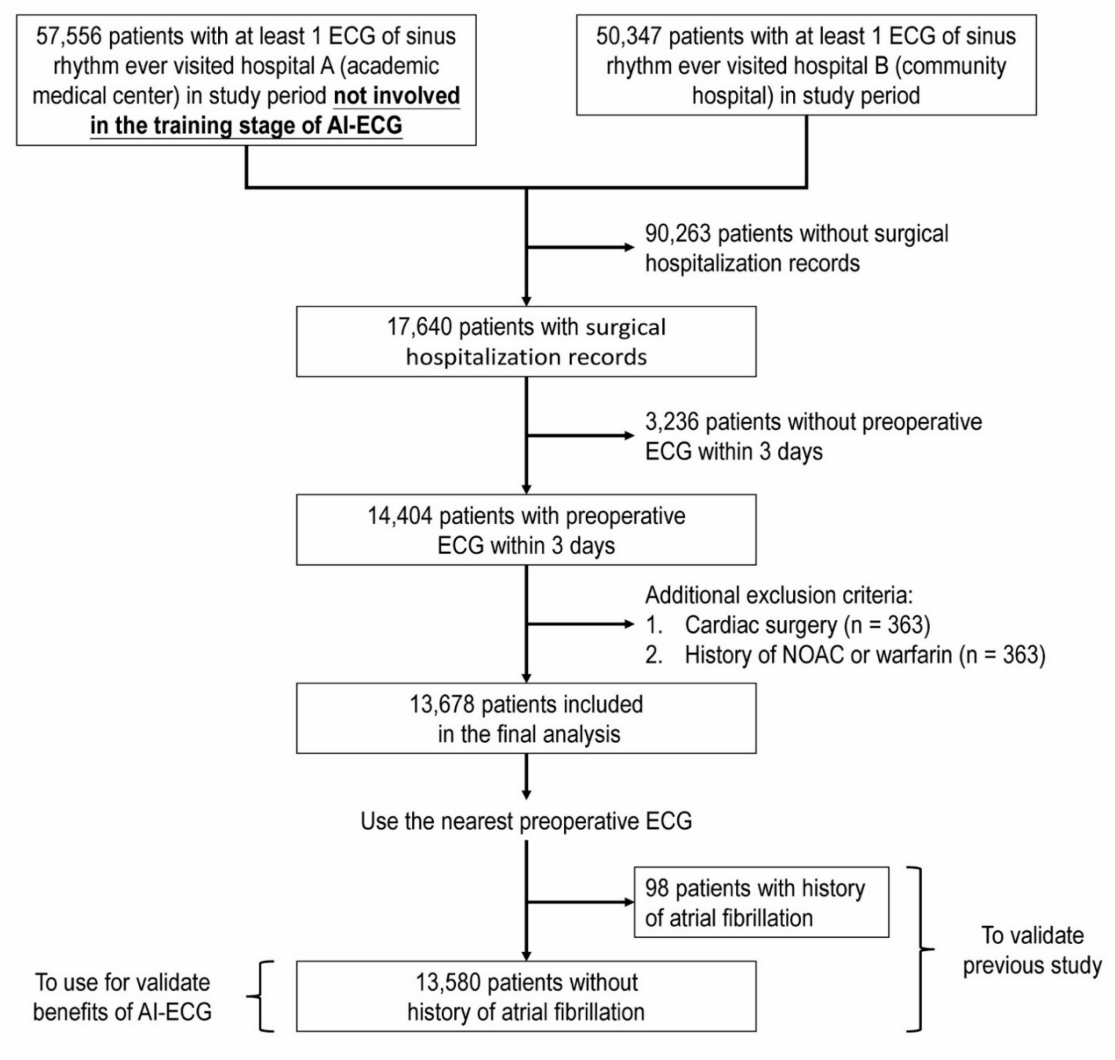
** Flow diagram.** The artificial intelligence (AI) model was trained using the dataset from Hospital A, with the remaining patients not involved in the training stage of the AI-electrocardiogram (ECG) analysis used for subsequent validation (details are provided in Supplementary Figure S1).

Figure 1 shows the flowchart for the selection of our study sample. Of the 107,903 screened patients, 17,640 had medical records of admission and had undergone surgery. After excluding 3,236 patients without preoperative ECGs within 3 days, 363 patients who underwent cardiac surgery, 363 patients who received anticoagulant treatment, 13,687 patients remained eligible for study analysis. According to our classification, the pre-AF group (untreated pre-AF before surgery) comprised 98 patients, the NOAF group (NOAF within 30 days post-surgery) comprised 54 patients, and the control group (all other patients) comprised 13,526 patients. Table 1 presents the baseline characteristics of the three groups. Patients in the control group were significantly younger, had lower AF stroke risk scores (CHA2DS2-VASc scores), and had fewer comorbidities.

Focusing on NOAF occurring within 30 days post-surgery, the overall incidence was 0.4% in our study. A detailed analysis, including surgical characteristics and stratified results, is summarized in Table 2. The incidence of NOAF was notably higher in the high-risk surgery group (0.7%) compared to the low-risk group (0.3%). Stratification by surgical specialty revealed that cardiovascular surgery (excluded cardiac surgery) had the highest NOAF incidence at 1.8%, followed by chest surgery at 1.2%. Additionally, within the NOAF cohort, early-onset NOAF (defined as ≤ 48 hours post-surgery) accounted for 40.7%, whereas late-onset NOAF constituted 59.3% (median 12 days; interquartile range 4.4-16.7 days).

### Perioperative Clinical Outcomes in a Population with Hidden AF

**Figure 2:**
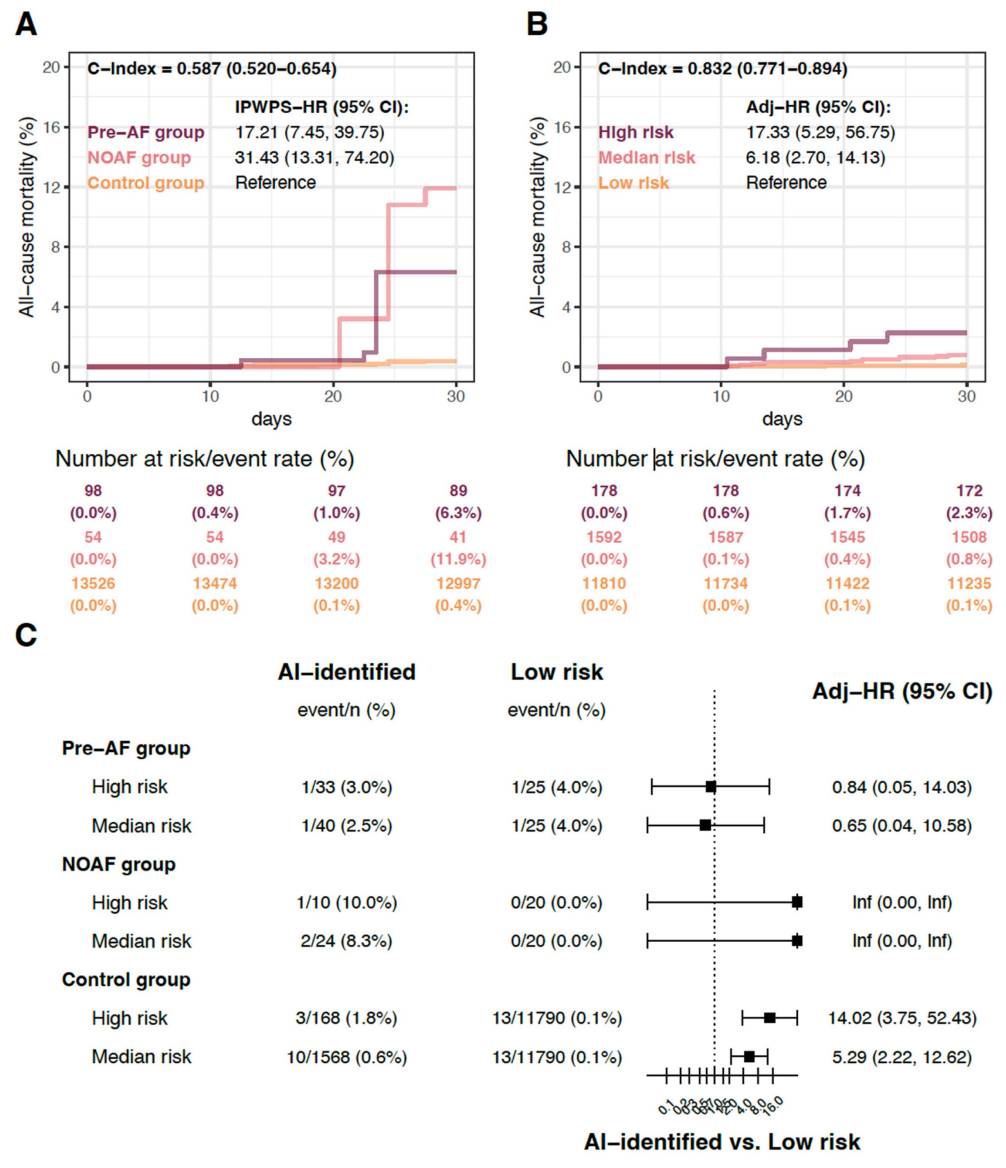
** Risk of all-cause mortality after surgery.** The AI-ECG-identified high-risk was defined by the cut-off point corresponding to a high positive predictive value (p > 0.994, Figure S3), while the medium-risk was defined by the cut-off point associated with high sensitivity (p > 0.047, Figure S2). (a) A comparison of patients with and without observed atrial fibrillation, involving 13,678 patients to validate a previous study. The hazard ratio (HR) was adjusted using inverse probability weighting of the propensity score (IPWPS). (b) The relationship between AI-ECG prediction and all-cause mortality in patients without a history of atrial fibrillation, including 13,580 patients, demonstrating the benefits of AI-ECG. HRs were adjusted for age and sex. (c) Stratified analysis of observed atrial fibrillation, with HRs adjusted for age and sex. Abbreviations: hx, history; AF, atrial fibrillation; HR, hazard ratio.

To balance baseline characteristic differences, we applied inverse probability weighting of propensity scores (IPWPS) for adjustment. Detailed process is described in the Supplementary Materials under the section “Propensity Score Modelling and Covariate Adjustment”. After adjustment, compared with the control group, the patients in pre-AF and NOAF groups had significantly higher 30-day all-cause mortality rates, with HRs of 17.21 (95% CI 7.45-39.75) and 31.43 (95% CI 13.31-74.20), respectively (Figure 2A). Sensitivity analysis with adjustments for only age and sex revealed that patients in the pre-AF and NOAF groups continued to show significantly higher 30-day all-cause mortality rates than those in the control group (Supplementary Figure S8). These findings support the clinical evidence that AF, whether in patients with a history of AF or those developing postoperative AF, significantly increases mortality risk.

### AI-ECG Identification of Hidden AF and Associated Perioperative Outcomes

We applied the AI-ECG model to all 13,580 patients without pre-existing AF (the NOAF and control groups) and stratified them into high-, medium-, and low-risk categories for hidden AF. There were 178 patients (1.3%) classified as high risk and 1,592 patients (11.7%) as medium-risk. In the high-risk group, 10 patients (5.6%) developed postoperative NOAF within one month. Among the remaining 168 high-risk patients without documented postoperative NOAF within one month, 26 patients (15.5%) were diagnosed with AF within one year, and an additional 19 patients (11.3%) were diagnosed thereafter. Detailed was shown in Table 3.

To validate the benefit of AI-ECG in patients unaware of their AF-related perioperative risk, we analyzed clinical outcomes stratified by the AI-ECG predictions. The 178 patients categorized in the high-risk group had significantly higher all-cause mortality rates than those in the low-risk group within 30 days postoperatively (HR 17.33, 95% CI 5.29-56.75; Figure 2B). The medium-risk group also exhibited significantly higher all-cause mortality rates than the low-risk group (HR 6.18, 95% CI 2.70-14.13). Figure 2C shows the comparison of the patients with different risk categories based on the AI-ECG predictions. Within the pre-AF and NOAF groups, no significant differences were observed between the high, medium and low risk subgroups. However, patients with high and medium risks in the control group showed significantly higher all-cause mortality rates than those in the low-risk group, with HRs of 14.02 (95% CI 3.75-52.43) and 5.29 (95% CI 2.22-12.62), respectively.

**Figure 3:**
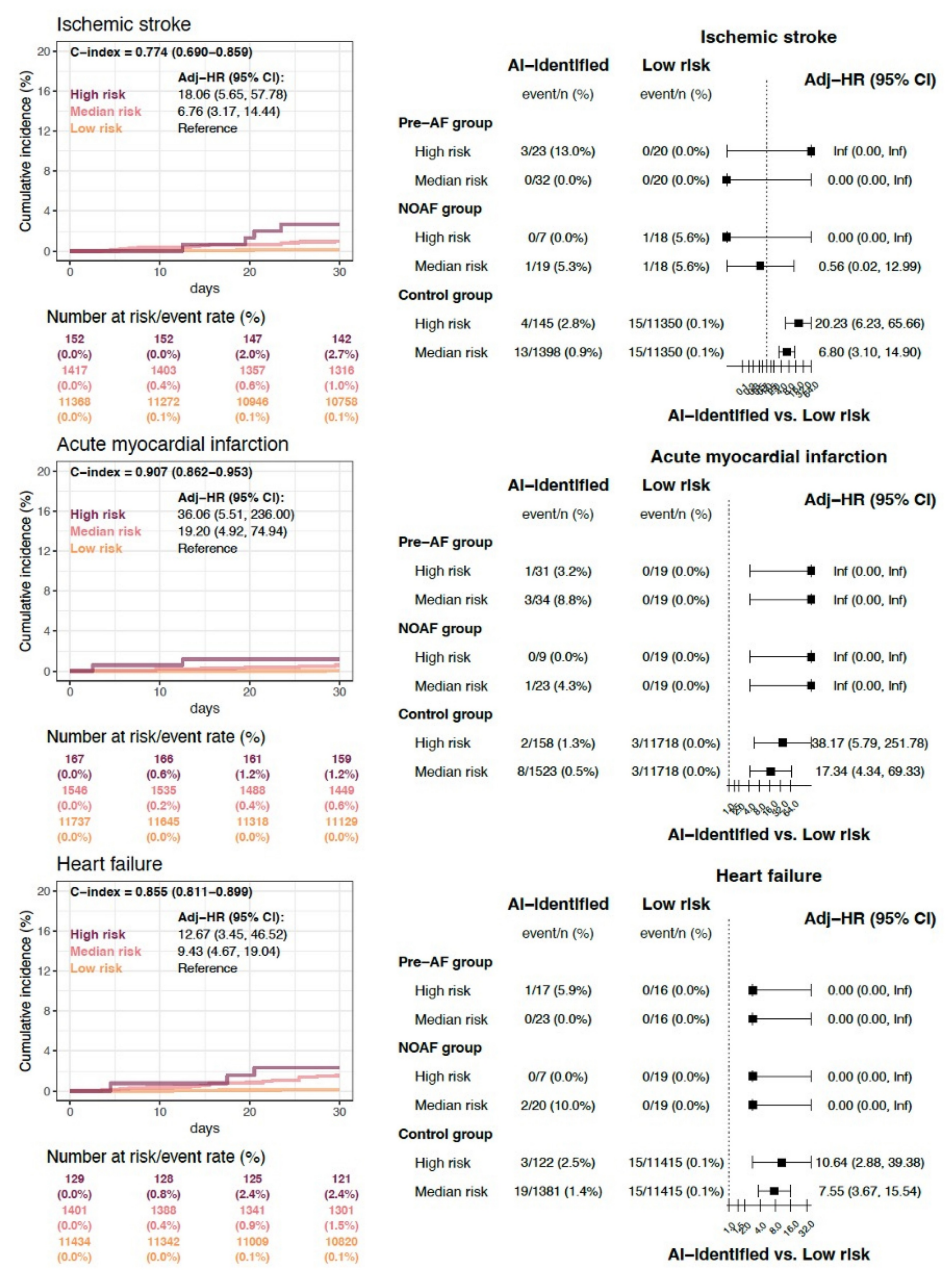
** Secondary clinical outcomes stratified by AI-ECG risk categories.** Individuals classified as high risk for hidden AF exhibited the highest cumulative incidence rates of new-onset ischemic stroke (HRs of 18.06; 95% CI 5.65 - 57.78), acute myocardial infarction (HRs of 36.06; 95% CI 5.51 - 236), and heart failure (HRs of 12.67; 95% CI 3.45 - 46.52) within 30 days post-surgery, followed by those at medium and low risk. The comparison of patients across different risk categories based on AI-ECG predictions is summarized in the right panel.

The secondary clinical outcomes stratified by AI-ECG risk categories are presented in Figure 3. Individuals classified as high risk for hidden AF exhibited the highest cumulative incidence rates of new-onset ischemic stroke, acute myocardial infarction, and heart failure within 30 days post-surgery, followed by those at medium and low risk. The comparison of patients across different risk categories based on AI-ECG predictions is summarized in the right panel of Figure 3. Consistent with the findings in Figure 2C, within the control group, patients classified as high and medium risk by AI-ECG also demonstrated significantly higher event rates of all secondary clinical outcomes compared to the low-risk group.

### Comparison of AI-ECG, C2HEST, and TWAFS

**Figure 4:**
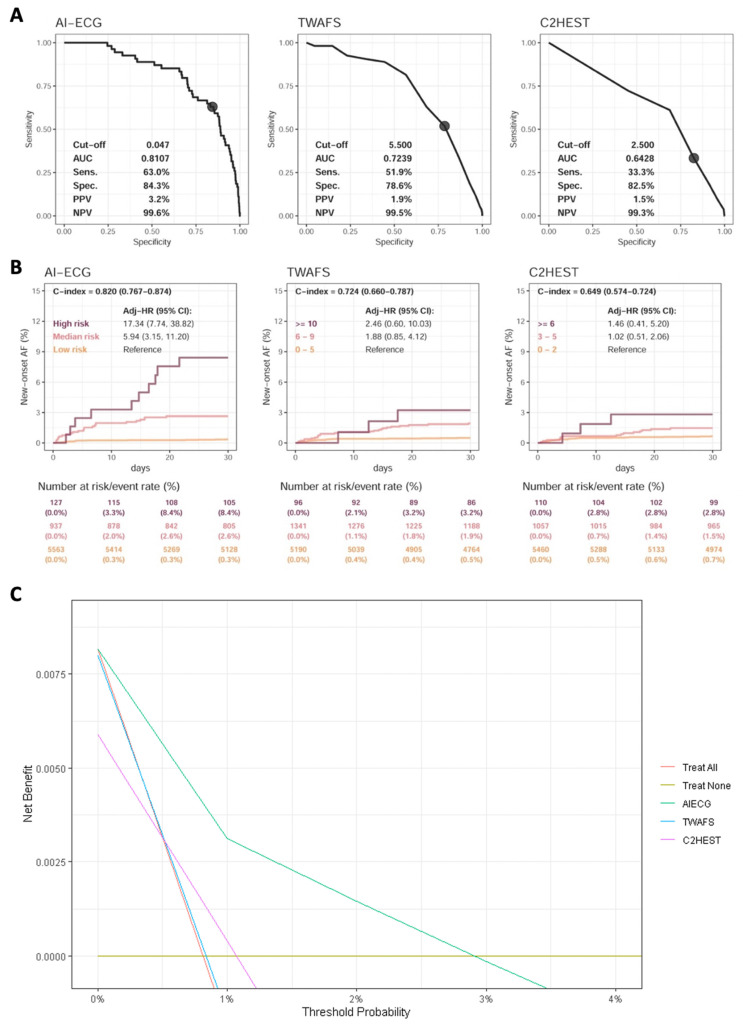
** Comparison of AI-ECG and clinical risk scores on identifying new-onset atrial fibrillation.** Analyses included the data of 13,580 patients without pre-existing atrial fibrillation to validate AI-ECG benefits. (a) Receiver operating characteristic curve analysis of postoperative atrial fibrillation within 1 month, with cut-off points for AI-ECG, Taiwan AF scores, and C2HEST set at 0.047 (defined in Figure S2), 5.5, and 2.5, respectively. (b) One-month follow-up analyses, where the AI-ECG-identified high risk was defined by a high positive predictive value cut-off (p > 0.994, Figure S3) and the medium-risk by a high sensitivity cut-off (p > 0.047, Figure S2). C-indexes were calculated using continuous scores. Abbreviations: AF, atrial fibrillation; HR, hazard ratio.

The performance of AI-ECG compared with other risk scores for predicting AF was showed in Figure 4A. A total of 13,580 patients without pre-AF in our study population were eligible for the analysis. After excluding patients without follow-up data within 1 month, AI-ECG demonstrated the highest performance, with an AUC of 0.8107, followed by TWAFS with an AUC of 0.7239, and C2HEST with an AUC of 0.6428 for predicting NOAF within 30 days post-surgery.

In Figure 4B, AI-ECG achieved the highest discrimination for NOAF within 30 days (C index 0.820; 95% CI 0.767-0.874), followed by TWAFS (C index 0.724; 95% CI 0.660-0.787) and C2HEST (C index 0.649; 95% CI 0.574-0.724). AI-ECG also clearly separated risk groups: high-risk patients experienced a 17-fold higher NOAF incidence (HR 17.34; 95% CI 7.74-38.82), and medium-risk patients a nearly six-fold higher incidence (HR 5.94; 95% CI 3.15-11.20), relative to low-risk individuals.

Decision curve analysis was performed to evaluate the clinical net benefit of the AI-ECG model compared to other models. (Figure 4C) The AI-ECG model demonstrated superior net benefit across most threshold probabilities compared to C2HEST and TWAFS. The increasing net benefit of AI-ECG at higher threshold probabilities reflects its stronger ability to distinguish high-risk patients. Furthermore, we assessed the predictive performance of TWAFS and C2HEST for short-term (30-day) postoperative mortality (Supplementary Figure S9). Both models exhibited modest discriminative ability, with C-index of 0.669 and 0.631, respectively, whereas the AI-ECG model achieved a substantially higher C-index of 0.832.

## Discussion

We developed an AI-ECG model to identify patients with hidden AF using SR-ECG without the need for additional clinical information. The model was validated on both internal and external datasets, demonstrating strong performance. The model demonstrated robust performance across both internal and external datasets. Importantly, our findings demonstrate that a high AI-ECG risk —although not directly trained on postoperative outcomes—can effectively stratify patients at increased risk of perioperative complications. Based on AI-ECG assessment of the perioperative risk of patients without pre-AF undergoing non-cardiac surgery, the high-risk population had significantly higher rates of all-cause mortality, new-onset ischemic stroke, new-onset acute myocardial infarction, and new-onset heart failure within 30 days postoperatively. This pioneering study represents the first use of AI-ECG to stratify hidden AF-related perioperative risks of patients undergoing non-cardiac surgery.

Since the Mayo Clinic team developed the first AI model to identify individuals with a high likelihood of AF through SR-ECG, several studies have reported consistent results.^25^, ^27^, ^28^, ^31^ Our study replicated the strong performance of the existing model, achieving 80% accuracy in detecting hidden AF in subgroups with pre-AF and NOAF. Several studies have shown that AI models perform comparably to the CHARGE-AF score in predicting long-term incident AF in Western populations.^25^, ^31^, ^32^ Our AI-ECG system was developed specifically for the Asian population. When compared with the Asian AF prediction score, C2HEST, and TWAFS, our AI-ECG model demonstrated significantly superior performance in predicting short-term NOAF. This finding reinforces the potential of AI to outperform traditional risk scores for predicting short-term AF events.^31^

The reported incidence of NOAF after non-cardiac surgery varies widely, ranging from 0.4% to 30%. In our study, the overall incidence was 0.4%, which is comparable to findings from other Asian countries but notably lower than those reported in Western cohorts.^33^-^36^ This discrepancy may be attributed to differences in patient demographics, surgery types, and population health profiles. In our study, the low incidence may partly reflect the inclusion of both major and minor surgeries; however, insufficient systematic screening and the resultant underdiagnosis are likely major contributors. Our results were consistent with those of previous studies indicating that hidden AF significantly increases short-term all-cause mortality and adverse events following non-cardiac surgery.^16^-^20^ These findings reinforce the clinical importance of early identification of patients at high risk of NOAF and the implementation of appropriate monitoring strategies to mitigate adverse outcomes.

Given the low overt incidence yet substantial clinical consequences of undiagnosed AF, a proactive identification strategy is warranted. Several factors make it challenging to recognize or manage AF-related perioperative risk in patients undergoing non-cardiac surgery. Applying our AI-ECG system in clinical practice allows for the identification and targeted management of patients at risk of AF-related perioperative complications during hospitalization. Additionally, it enables the recognition and monitoring of patients with a high probability of developing AF over long-term follow-up.

The short-term mortality rate after non-cardiac surgery is approximately 1.2-1.5%.^37^, ^38^ Numerous predictor scores have been developed to mitigate surgical risks and mortality.^39^-^41^ However, each of these scoring systems has limitations, such as reliance on subjective intuition, complexity in application, or suboptimal predictive performance. Recently, several DLMs that accurately predict postoperative mortality have been introduced. One such model leverages objective, quantitative data from electronic medical record systems to predict 30-day mortality after non-cardiac surgery and has demonstrated potential for implementation across multiple hospitals.^42^ Another model showed exceptional performance in discriminating postoperative mortality using a single perioperative ECG.^43^ Although these models effectively identify high-risk populations, they do not provide insights into the exact causes of mortality or specific prevention strategies. While correcting objective data may help reduce short-term mortality, its efficacy remains uncertain. Our study utilized AI-ECG to detect hidden AF and assess perioperative risks linked to AF. This research also established a correlation between hidden AF and postoperative mortality, as well as various comorbidities. Notably, the model may capture not only AF-specific electrical patterns but also broader cardiovascular vulnerability related to AF development, which may overlap with factors contributing to elevated postoperative risk. Understanding the risks associated with AF itself or AF-driven risk still enables physicians to focus on targeted management, enhancing clinical accuracy, and improving patient outcomes.

This study had several limitations. First, all patients were diagnosed with AF using a 12-lead ECG rather than continuous monitoring, making the underdiagnosis of AF unavoidable. Second, postoperative ECGs were not routinely performed in our hospitals. Patients who did undergo ECGs likely had symptoms or specific clinical indications, which could introduce selection bias. Those who were asymptomatic or lacked clinical indications had a high probability of missed diagnoses. It may also explain why all clinical outcomes were significantly worse in the AI-ECG high-risk group compared to the low-risk group in control group. Third, the AI-ECG model was not prospectively validated prior to its application. Although we conducted internal and external validations and obtained results consistent with previous studies, the accuracy of the model may not be fully optimal. Prospective research would help address these limitations, and this is a planned focus for future work.

## Conclusion

We developed an AI-ECG model to accurately predict hidden AF (pre-AF and NOAF within 30 days) using a single SR-ECG. The model effectively stratifies AF-related perioperative risks, predicting 30-day all-cause mortality and new-onset ischemic stroke, acute myocardial infarction, and heart failure in individuals undergoing non-cardiac surgery.

## Supplementary Material

Supplementary materials and methods, figures and tables.

## Figures and Tables

**Table 1:** Baseline characteristics stratified by observed atrial fibrillation.

	Pre-existing AF (Pre-AF Group, n = 98)	New-onset AF within 30 days after operation (NOAF Group, n = 54)	Other patients (Control Group, n = 13526)	p-value
Surgery type*				< 0.001
High risk	37 (37.8%)	17 (31.5%)	2556 (18.9%)	
Low risk	61 (62.2%)	37 (68.5%)	10970 (81.1%)	
Hospital				0.487
Academic medical center	68 (69.4%)	42 (77.8%)	9531 (70.5%)	
Community hospital	30 (30.6%)	12 (22.2%)	3995 (29.5%)	
Sex (male)	61 (62.2%)	32 (59.3%)	6385 (47.2%)	0.003
Age (y/o, mean ± SD)	71.8±12.4	70.0±13.2	58.2±14.7	< 0.001
CHA_2_DS_2_-VASc (mean ± SD)	4.5±2.0	3.2±2.0	1.8±1.7	< 0.001
CHA_2_DS_2_-VASc group				< 0.001
0	3 (3.1%)	2 (3.7%)	2683 (19.8%)	
1	2 (2.0%)	12 (22.2%)	4668 (34.5%)	
2	9 (9.2%)	7 (13.0%)	2425 (17.9%)	
3	18 (18.4%)	12 (22.2%)	1632 (12.1%)	
4	21 (21.4%)	8 (14.8%)	1040 (7.7%)	
5	13 (13.3%)	5 (9.3%)	560 (4.1%)	
6	17 (17.3%)	6 (11.1%)	303 (2.2%)	
7-9	15 (15.3%)	2 (3.7%)	215 (1.6%)	
RCRI (mean ± SD)	2.0±1.1	1.3±1.2	0.5±0.8	< 0.001
RCRI group				< 0.001
0	5 (5.1%)	17 (31.5%)	8615 (63.7%)	
1	31 (31.6%)	18 (33.3%)	3675 (27.2%)	
2	30 (30.6%)	10 (18.5%)	893 (6.6%)	
3	23 (23.5%)	6 (11.1%)	266 (2.0%)	
4-5	9 (9.2%)	3 (5.6%)	77 (0.6%)	
Diabetes mellitus	51 (52.0%)	17 (31.5%)	2897 (21.4%)	< 0.001
Diabetes mellitus requiring insulin	13 (13.3%)	3 (5.6%)	404 (3.0%)	< 0.001
Serum creatinine ≥ 2 mg/dL	55 (56.1%)	17 (31.5%)	846 (6.3%)	< 0.001
End stage renal disease	46 (46.9%)	11 (20.4%)	637 (4.7%)	< 0.001
Hypertension	79 (80.6%)	23 (42.6%)	4303 (31.8%)	< 0.001
Coronary artery disease	60 (61.2%)	15 (27.8%)	1600 (11.8%)	< 0.001
Peripheral arterial occlusion disease	22 (22.4%)	1 (1.9%)	332 (2.5%)	< 0.001
Heart failure	42 (42.9%)	8 (14.8%)	608 (4.5%)	< 0.001
Transient ischemic attack	9 (9.2%)	3 (5.6%)	450 (3.3%)	0.007
Ischemic stroke	23 (23.5%)	10 (18.5%)	633 (4.7%)	< 0.001
Hemorrhagic stroke	13 (13.3%)	4 (7.4%)	385 (2.8%)	< 0.001
Chronic obstructive pulmonary disease	30 (30.6%)	7 (13.0%)	1250 (9.2%)	< 0.001
Alcoholism	3 (3.1%)	4 (7.4%)	271 (2.0%)	0.023

* High-risk surgery is defined as major vascular, intraperitoneal, and intrathoracic surgeries. Surgeries not meeting these criteria are classified as low-risk. The p-value was two-sided, with no adjustment for multiple comparison. Abbreviations: AF, atrial fibrillation; RCRI, revised cardiac risk index; SD, standard deviation.

**Table 2:** Incidence of Post-operative NOAF within 30 Days and Surgical Characteristics

	Total Patient Number (n=13678)	New-onset AF within 30 days after operation (NOAF Group, n = 54)	Incidence of NOAF
Surgery type*			
High risk	2610 (19.1%)	17 (31.5%)	0.7%
Low risk	11068 (80.9%)	37 (68.5%)	0.3%
Surgical Specialty			
Cardiovascular Surgery	399 (2.9%)	7 (13.0%)	1.8%
Chest Surgery	696 (5.1%)	8 (14.8%)	1.2%
Plastic Surgery	839 (6.1%)	5 (9.3%)	0.6%
Neurosurgery	2615 (19.1%)	15 (27.8%)	0.6%
General Surgery	2329 (17.0%)	12 (22.2%)	0.5%
Orthopedics	2689 (19.7%)	5 (9.3%)	0.2%
Gynecology	1169 (8.6%)	1 (1.9%)	0.1%
Genitourinary Surgery	1351 (9.9%)	1 (1.9%)	0.1%
Other surgery	1591 (11.6%)	0 (0%)	0.1%

Other surgeries included specialties of the Ear, Nose, and Throat, Ophthalmology, and Oral and Maxillofacial Surgery. * High-risk surgery is defined as major vascular, intraperitoneal, and intrathoracic surgeries. Surgeries not meeting these criteria are classified as low-risk.

**Table 3:** AI-ECG Identification of Postoperative NOAF

	Total Patients without pre-existing AF (n = 13580)	New-onset AF within 30 days after surgery	New-onset AF within 1 year (30 days to 1 year)	New-onset AF after 1 year
High risk	178 (178/13580; 1.3%)	10 (10/178; 5.6%)	26 (26/168; 15.5%)	19 (19/142; 13.4%)
Medium risk	1592 (1592/13580; 11.7%)	24 (1.5%)	-	-
Low risk	11810 (11810/13580; 87%)	20 (0.2%)	-	-

## Abbreviations

AI-ECG: Artificial Intelligence-Enabled Electrocardiography
AF: Atrial Fibrillation
NOAF: New-Onset Atrial Fibrillation
DLM: Deep Learning Model
SR-ECG: Sinus Rhythm Electrocardiogram
ROC: Receiver Operating Characteristic
PRROC: Precision-Recall Receiver Operating Characteristic
IPWPS: Inverse Probability Weighting of the Propensity Score
RCRI: Revised Cardiac Risk Index
TWAFS: Taiwan AF Scores
HR: Hazard Ratio
CI: Confidence Interval
SD: Standard Difference
SMD: Standardized Mean Difference

## References

[1] Chao TF, Liu CJ, Tuan TC, Chen TJ, Hsieh MH, Lip GYH (2018). Lifetime Risks, Projected Numbers, and Adverse Outcomes in Asian Patients With Atrial Fibrillation: A Report From the Taiwan Nationwide AF Cohort Study. Chest.

[2] Gladstone DJ, Spring M, Dorian P, Panzov V, Thorpe KE, Hall J (2014). Atrial fibrillation in patients with cryptogenic stroke. N Engl J Med.

[3] Sanna T, Diener HC, Passman RS, Di Lazzaro V, Bernstein RA, Morillo CA (2014). Cryptogenic stroke and underlying atrial fibrillation. N Engl J Med.

[4] Jayachandran JV, Sih HJ, Winkle W, Zipes DP, Hutchins GD, Olgin JE (2000). Atrial fibrillation produced by prolonged rapid atrial pacing is associated with heterogeneous changes in atrial sympathetic innervation. Circulation.

[5] Allessie M, Ausma J, Schotten U (2002). Electrical, contractile and structural remodeling during atrial fibrillation. Cardiovasc Res.

[6] Brundel BJ, Henning RH, Kampinga HH, Van Gelder IC, Crijns HJ (2002). Molecular mechanisms of remodeling in human atrial fibrillation. Cardiovasc Res.

[7] Wijffels MC, Kirchhof CJ, Dorland R, Allessie MA (1995). Atrial fibrillation begets atrial fibrillation. A study in awake chronically instrumented goats. Circulation.

[8] Liao CT, Lee MC, Chen ZC, Ku LE, Wang JD, Toh HS (2020). Cost-Effectiveness Analysis of Oral Anticoagulants in Stroke Prevention among Patients with Atrial Fibrillation in Taiwan. Acta Cardiol Sin.

[9] Gunawardene MA, Willems S (2022). Atrial fibrillation progression and the importance of early treatment for improving clinical outcomes. Europace.

[10] Kirchhof P, Camm AJ, Goette A, Brandes A, Eckardt L, Elvan A (2020). Early Rhythm-Control Therapy in Patients with Atrial Fibrillation. N Engl J Med.

[11] Turakhia MP, Shafrin J, Bognar K, Trocio J, Abdulsattar Y, Wiederkehr D (2018). Estimated prevalence of undiagnosed atrial fibrillation in the United States. PLoS One.

[12] Goette A, Kalman JM, Aguinaga L, Akar J, Cabrera JA, Chen SA (2016). EHRA/HRS/APHRS/SOLAECE expert consensus on atrial cardiomyopathies: definition, characterization, and clinical implication. Europace.

[13] Staerk L, Sherer JA, Ko D, Benjamin EJ, Helm RH (2017). Atrial Fibrillation: Epidemiology, Pathophysiology, and Clinical Outcomes. Circ Res.

[14] Nattel S, Burstein B, Dobrev D (2008). Atrial remodeling and atrial fibrillation: mechanisms and implications. Circ Arrhythm Electrophysiol.

[15] De Jong AM, Maass AH, Oberdorf-Maass SU, Van Veldhuisen DJ, Van Gilst WH, Van Gelder IC (2011). Mechanisms of atrial structural changes caused by stretch occurring before and during early atrial fibrillation. Cardiovasc Res.

[16] Lin MH, Kamel H, Singer DE, Wu YL, Lee M, Ovbiagele B (2019). Perioperative/Postoperative Atrial Fibrillation and Risk of Subsequent Stroke and/or Mortality. Stroke.

[17] van Diepen S, Bakal JA, McAlister FA, Ezekowitz JA (2011). Mortality and readmission of patients with heart failure, atrial fibrillation, or coronary artery disease undergoing noncardiac surgery: an analysis of 38 047 patients. Circulation.

[18] Prasada S, Desai MY, Saad M, Smilowitz NR, Faulx M, Menon V (2022). Preoperative Atrial Fibrillation and Cardiovascular Outcomes After Noncardiac Surgery. J Am Coll Cardiol.

[19] Brathwaite D, Weissman C (1998). The new onset of atrial arrhythmias following major noncardiothoracic surgery is associated with increased mortality. Chest.

[20] Polanczyk CA, Goldman L, Marcantonio ER, Orav EJ, Lee TH (1998). Supraventricular arrhythmia in patients having noncardiac surgery: clinical correlates and effect on length of stay. Ann Intern Med.

[21] Chao TF, Chiang CE, Chen TJ, Liao JN, Tuan TC, Chen SA (2021). Clinical Risk Score for the Prediction of Incident Atrial Fibrillation: Derivation in 7 220 654 Taiwan Patients With 438 930 Incident Atrial Fibrillations During a 16-Year Follow-Up. J Am Heart Assoc.

[22] Li YG, Pastori D, Farcomeni A, Yang PS, Jang E, Joung B (2019). A Simple Clinical Risk Score (C(2)HEST) for Predicting Incident Atrial Fibrillation in Asian Subjects: Derivation in 471,446 Chinese Subjects, With Internal Validation and External Application in 451,199 Korean Subjects. Chest.

[23] Alonso A, Krijthe BP, Aspelund T, Stepas KA, Pencina MJ, Moser CB (2013). Simple risk model predicts incidence of atrial fibrillation in a racially and geographically diverse population: the CHARGE-AF consortium. J Am Heart Assoc.

[24] Schnabel RB, Sullivan LM, Levy D, Pencina MJ, Massaro JM, D'Agostino RB Sr (2009). Development of a risk score for atrial fibrillation (Framingham Heart Study): a community-based cohort study. Lancet.

[25] Raghunath S, Pfeifer JM, Ulloa-Cerna AE, Nemani A, Carbonati T, Jing L (2021). Deep Neural Networks Can Predict New-Onset Atrial Fibrillation From the 12-Lead ECG and Help Identify Those at Risk of Atrial Fibrillation-Related Stroke. Circulation.

[26] Melzi P, Tolosana R, Cecconi A, Sanz-Garcia A, Ortega GJ, Jimenez-Borreguero LJ (2021). Analyzing artificial intelligence systems for the prediction of atrial fibrillation from sinus-rhythm ECGs including demographics and feature visualization. Sci Rep.

[27] Attia ZI, Noseworthy PA, Lopez-Jimenez F, Asirvatham SJ, Deshmukh AJ, Gersh BJ (2019). An artificial intelligence-enabled ECG algorithm for the identification of patients with atrial fibrillation during sinus rhythm: a retrospective analysis of outcome prediction. Lancet.

[28] Noseworthy PA, Attia ZI, Behnken EM, Giblon RE, Bews KA, Liu S (2022). Artificial intelligence-guided screening for atrial fibrillation using electrocardiogram during sinus rhythm: a prospective non-randomised interventional trial. Lancet.

[29] Lip GY, Nieuwlaat R, Pisters R, Lane DA, Crijns HJ (2010). Refining clinical risk stratification for predicting stroke and thromboembolism in atrial fibrillation using a novel risk factor-based approach: the euro heart survey on atrial fibrillation. Chest.

[30] Fleisher LA, Fleischmann KE, Auerbach AD, Barnason SA, Beckman JA, Bozkurt B (2014). 2014 ACC/AHA guideline on perioperative cardiovascular evaluation and management of patients undergoing noncardiac surgery: executive summary: a report of the American College of Cardiology/American Heart Association Task Force on Practice Guidelines. Circulation.

[31] Khurshid S, Friedman S, Reeder C, Di Achille P, Diamant N, Singh P (2022). ECG-Based Deep Learning and Clinical Risk Factors to Predict Atrial Fibrillation. Circulation.

[32] Christopoulos G, Graff-Radford J, Lopez CL, Yao X, Attia ZI, Rabinstein AA (2020). Artificial Intelligence-Electrocardiography to Predict Incident Atrial Fibrillation: A Population-Based Study. Circ Arrhythm Electrophysiol.

[33] Sohn GH, Shin DH, Byun KM, Han HJ, Cho SJ, Song YB (2009). The incidence and predictors of postoperative atrial fibrillation after noncardiothoracic surgery. Korean Circ J.

[34] Hyun J, Cho MS, Nam GB, Kim M, Do U, Kim J (2021). Natural Course of New-Onset Postoperative Atrial Fibrillation after Noncardiac Surgery. J Am Heart Assoc.

[35] Jiang S, Liao X, Chen Y, Li B (2023). Exploring postoperative atrial fibrillation after non-cardiac surgery: mechanisms, risk factors, and prevention strategies. Front Cardiovasc Med.

[36] Wang MK, Razeghi G, Baskaran G, Park L, Blum S, Heo R (2025). Rhythm vs Rate Control Strategies for Perioperative Atrial Fibrillation After Noncardiac Surgery: A Systematic Review and Meta-analysis. CJC Open.

[37] Smilowitz NR, Gupta N, Ramakrishna H, Guo Y, Berger JS, Bangalore S (2017). Perioperative Major Adverse Cardiovascular and Cerebrovascular Events Associated with Noncardiac Surgery. JAMA Cardiol.

[38] Devereaux PJ, Sessler DI (2015). Cardiac Complications in Patients Undergoing Major Noncardiac Surgery. N Engl J Med.

[39] Gawande AA, Kwaan MR, Regenbogen SE, Lipsitz SA, Zinner MJ (2007). An Apgar score for surgery. J Am Coll Surg.

[40] Bilimoria KY, Liu Y, Paruch JL, Zhou L, Kmiecik TE, Ko CY (2013). Development and evaluation of the universal ACS NSQIP surgical risk calculator: a decision aid and informed consent tool for patients and surgeons. J Am Coll Surg.

[41] Cohen ME, Bilimoria KY, Ko CY, Richards K, Hall BL (2009). Effect of subjective preoperative variables on risk-adjusted assessment of hospital morbidity and mortality. Ann Surg.

[42] Lee SW, Lee HC, Suh J, Lee KH, Lee H, Seo S (2022). Multi-center validation of machine learning model for preoperative prediction of postoperative mortality. NPJ Digit Med.

[43] Ouyang D, Theurer J, Stein NR, Hughes JW, Elias P, He B (2024). Electrocardiographic deep learning for predicting post-procedural mortality: a model development and validation study. Lancet Digit Health.

