# Supplementary material for: Artificial Intelligence-Enabled Electrocardiography for Preoperatively Detecting Atrial Fibrillation and Mortality Risk in Patients with Sinus Rhythm
Source: Int J Med Sci. 2026 Jan 14;23(2):684–94. doi: 10.7150/ijms.123598 (PMC12825122; doi:10.7150/ijms.123598)
Supplement: Supplementary file 1 — Supplementary materials and methods, figures and tables. [file ijmsv23p0684s1.pdf]

Supplemental Materials

1. Supplemental Manuscript

AI-ECG Development and Validation

AI-ECG Model Performance

AI-ECG Model Characterization and Explainability Analyses

Propensity Score Modelling and Covariate Adjustment

Supplemental manuscript reference

2. Supplemental Tables

Table S1 | Corresponding patient characteristics among each dataset in the training stage of AI-ECG.

Table S2 | Corresponding patient characteristics among Internal and External Validation dataset.

Table S3 | The logistic regression coefficients of propensity score model.

Table S4 | Baseline characteristics stratified by observed atrial fibrillation after inverse probability weighting.

3. Supplemental Figures

Figure S1 | Flow diagram for AI-ECG development.

Figure S2 | The ROC curve of AI-ECG predictions to detect paroxysmal atrial fibrillation.

Figure S3 | The PRROC curve of AI-ECG predictions to detect paroxysmal atrial fibrillation.

Figure S4 | Calibration Curves for AI-ECG Model Performance

Figure S5 | The interval between Sinus rhythm ECG and atrial fibrillation ECG.

Figure S6 | Correlation of ECG Features with AI-ECG Model Predictions

Figure S7 | The distribution of propensity score before and after inverse probability weighting.

Figure S8 | The comparison between patients with and without observed atrial fibrillation adjusted by age and sex on all-cause mortality.

Figure S9 | The relationship between clinical scores and all-cause mortality.

AI-ECG Development and Validation

We developed a deep learning model (DLM), referred to as the AI-ECG, to detect hidden atrial fibrillation (AF) using a 12-lead sinus rhythm (SR) electrocardiogram (ECG) without the need for additional patient information. Figure S1A shows patient diagnoses based on the timeline of the index SR-ECG and AF ECG. Hidden AF was the composite of: pre-existing AF (Pre-AF), defined as patients with a history of AF who were currently restored to SR, and new-onset AF (NOAF), defined as first-time recorded AF within 30 days following the index SR-ECG.

All adults (aged ≥18 years) who underwent at least two 10-second 12-lead ECGs at the Tri-Service General Hospital between August 1, 2013, and December 31, 2022, and had at least one ECG demonstrating SR were included in the model development. The model was fully retrained on 10-second, 12-lead SR-ECGs using the ECG12Net architecture, a DLM comprising 82 convolutional layers optimized for temporal-spatial ECG feature extraction, as detailed in prior research.[1] Among the screened 229,007 patients, there were 197,457 patients met our criteria, including 2,573 patients had at least a SR-ECG and an AF ECG.

The cohort data were divided into development, tuning, and internal validation sets in ratios of 50%, 20%, and 30%, respectively. Among the AF patients in development and tuning sets, 67% were pre-AF and 33% were NOAF cases. On average, 4.32 SR-ECGs per patient in the Pre-AF setting and 1.91 per patient in the NOAF setting were used for model training. To address class imbalance between Pre-AF and NOAF, we implemented random oversampling of the NOAF group during model training to equalize the number of samples in each groups. Validation was conducted using both internal and external datasets, with the external dataset obtained from the Tingjhou Branch of Tri-Service General Hospital under identical enrolment criteria. Details of the flow diagram are provided in Figure S1B. The baseline patients characteristics in each dataset are illustrated in Table S1.

All ECGs were recorded at a 500 Hz frequency with a 10-second duration per lead using a Philips 12-lead ECG machine (PH080A). Initial processing employed the Philips DXL ECG Algorithm, and all diagnoses were confirmed by cardiologists. Given the concurrent presentation of AF and atrial flutter and the shared treatment protocols, they were collectively classified as AF in this study.[2,3] All ECGs identified with AF were re-evaluated by additional cardiologists before inclusion. An expert committee comprising two electrophysiologists reviewed

ambiguous ECG cases, and eight cardiologists corroborated all AF diagnoses.

AI-ECG Model Performance

The performance of the AI-ECG model was evaluated using both the Receiver Operating Characteristic (ROC) and Precision-Recall (PR) curves across internal and external validation cohorts. The baseline characteristics and underlying comorbidities significantly differed between the internal and external validation cohorts, as detailed in Table S2. Consequently, these findings confirmed the critical role of external validation in assessing the model's generalizability across diverse patient populations.

As shown in Figure S2, the AI-ECG model demonstrated excellent performance in detecting hidden AF, pre-AF, and NOAF, with AUCs ranging from 0.87 to 0.88, 0.87, and 0.89 to 0.91, respectively, in both datasets. Correspondingly, the PR curves showed relatively modest PRAUC values, reflecting the difficulty in detecting positive cases due to the low prevalence of AF in the dataset (Figure S3). To further validate the model's predictive accuracy, calibration curves assessing the agreement between predicted probabilities and observed event rates were constructed. As presented in Figure S4, these calibration plots demonstrated good concordance across both internal and external validation cohorts, with consistent performance across hidden AF, pre-AF, and NOAF. Notably, calibration performance was particularly accurate at higher predicted risk levels, indicating superior model performance in identifying patients at elevated risk.

Based on these performance characteristics, two cutoff values were selected to stratify risk levels: a medium-risk cutoff of 0.047 and a high-risk cutoff of 0.994. The medium-risk cutoff balances sensitivity and specificity, with sensitivity around 70%-80% and specificity of 85%-86%, minimizing missed cases and tolerating some false positives. The high-risk cutoff is characterized by markedly lower sensitivity (approximately 26%-30%) but extremely high specificity (>98%) and near-perfect negative predictive value (NPV ~99.5%-100%). This cutoff emphasizes maximizing diagnostic certainty among those identified as high risk. These cutoff thresholds facilitate clinical risk stratification, allowing the model to identify patients at varying risk levels for AF with a balanced consideration of false positives and false negatives.

AI-ECG Model Characterization and Explainability Analyses

We provide the temporal relationship between SR and AF ECGs used for model training and validation in Figure S5. The pre-AF group exhibited a longer and more variable interval between the AF ECG and the SR index ECG in both datasets. In contrast, the NOAF group showed a shorter interval from the SR index ECG to the first AF detection. Despite these temporal differences, the AI-ECG model demonstrated consistent predictive performance across all groups (Figures S2–S4). Model performance was unaffected by the length of the interval between SR and AF ECG recordings.

To better understand the contributions of individual ECG features to the model's predictions, we conducted a feature–prediction correlation analysis. The results are shown in Figure S6, which illustrates the correlations between ECG features and the AI-ECG model’s predicted risk scores in both internal and external datasets. Features such as lower heart rate, prolonged QT and QTc intervals, longer QRS duration, and shortened PR interval showed consistently positive correlations across both datasets, suggesting stable contributions to the model’s risk estimation. In contrast, the correlations involving various ECG axes demonstrated inconsistency between the two cohorts. This inconsistency might result from cohort-specific differences in the ECG datasets or patient characteristics, indicating that axis-related features have limited contribution to the model’s generalizable AF prediction.

Propensity Score Modelling and Covariate Adjustment

Logistic regression coefficients for the propensity score models are presented in Table S3, separately for cases with pre-AF and postoperative NOAF within 1 month. Age, male sex, and Revised Cardiac Risk Index (RCRI) were consistently associated with higher AF risk in both models, whereas surgery risk type showed a negative association. The CHA2DS2-VASc score demonstrated a positive association in the pre-existing AF group but a negative association in the NOAF group.

As detailed in Table S4, Inverse Probability Weighting of the Propensity Score (IPWPS) substantially reduced baseline differences, achieving comparable distributions of propensity scores across groups. The standardized mean differences for surgery type, sex, age, CHA2DS2-VASc score, and RCRI were all below 0.2, which was considered acceptable balance for the study. After using of IPWPS effectively balanced these groups, the distribution of propensity scores appearing similar post-adjustment (Figure S7).

Supplemental manuscript reference

1. Lin CS, Lin C, Fang WH, Hsu CJ, Chen SJ, Huang KH, Lin WS, Tsai CS, Kuo CC, Chau T, et al. A Deep-Learning Algorithm (ECG12Net) for Detecting Hypokalemia and Hyperkalemia by Electrocardiography: Algorithm Development. JMIR Med Inform. 2020;8:e15931. doi: 10.2196/15931
2. Hindricks G, Potpara T, Dagres N, Arbelo E, Bax JJ, Blomström-Lundqvist C, Boriani G, Castella M, Dan GA, Dilaveris PE, et al. 2020 ESC Guidelines for the diagnosis and management of atrial fibrillation developed in collaboration with the European Association for Cardio-Thoracic Surgery (EACTS): The Task Force for the diagnosis and management of atrial fibrillation of the European Society of Cardiology (ESC) Developed with the special contribution of the European Heart Rhythm Association (EHRA) of the ESC. Eur Heart J. 2021;42:373-498. doi: 10.1093/eurheartj/ehaa612
3. Joglar JA, Chung MK, Armbruster AL, Benjamin EJ, Chyou JY, Cronin EM, Deswal A, Eckhardt LL, Goldberger ZD, Gopinathannair R, et al. 2023 ACC/AHA/ACCP/HRS Guideline for the Diagnosis and Management of Atrial Fibrillation: A Report of the American College of Cardiology/American Heart Association Joint Committee on Clinical Practice Guidelines. Circulation. 2024;149:e1-e156. doi: 10.1161/cir.0000000000001193

Table S1 | Corresponding patient characteristics among each dataset in the training stage of AI-ECG.

	Development (n = 101,233)	Tuning (n = 38,668)	Internal validation (n = 57,556)	External validation (n = 50,347)
**Department**				
Outpatient department	31841(31.5%)	12203(31.6%)	18036(31.3%)	22572(44.8%)
Emergency department	32221(31.8%)	12352(31.9%)	18112(31.5%)	14981(29.8%)
Inpatient department	26917(26.6%)	10274(26.6%)	15581(27.1%)	9285(18.4%)
Health check center	7461(7.4%)	2836(7.3%)	4295(7.5%)	1738(3.5%)
Unknown	2793(2.8%)	1003(2.6%)	1532(2.7%)	1771(3.5%)
**Sex (male)**	50832(50.2%)	19160(49.6%)	28279(49.1%)	24563(48.8%)
**Age (y/o, mean±SD)**	53.9±18.4	53.4±18.2	53.4±18.1	55.5±18.7
**CHA_2DS_2-VASc (mean±SD)**	1.7±1.7	1.7±1.6	1.7±1.6	2.1±1.9
**CHA_2DS_2-VASc group**				
0	23881(23.6%)	9322(24.1%)	13660(23.7%)	9177(18.2%)
1	35456(35.0%)	13614(35.2%)	20720(36.0%)	15425(30.6%)
2	16082(15.9%)	6234(16.1%)	9292(16.1%)	8772(17.4%)
3	10952(10.8%)	4118(10.6%)	5954(10.3%)	6534(13.0%)
4	6800(6.7%)	2535(6.6%)	3663(6.4%)	4450(8.8%)
5	4054(4.0%)	1451(3.8%)	2183(3.8%)	2805(5.6%)
6	2204(2.2%)	790(2.0%)	1189(2.1%)	1652(3.3%)
7-9	1804(1.8%)	604(1.6%)	895(1.6%)	1532(3.0%)
**TWAFS (mean±SD)**	1.9±3.2	1.8±3.1	1.8±3.1	2.4±3.3
**TWAFS group**				
0-5	84979(83.9%)	32897(85.1%)	48954(85.1%)	40224(79.9%)
6-9	14883(14.7%)	5346(13.8%)	7983(13.9%)	9162(18.2%)

	Development (n = 101,233)	Tuning (n = 38,668)	Internal validation (n = 57,556)	External validation (n = 50,347)
≥10	1371(1.4%)	425(1.1%)	619(1.1%)	961(1.9%)
**C2HEST (mean±SD)**	0.9±1.4	0.9±1.3	0.9±1.3	1.2±1.5
**C2HEST group**				
0-2	88910(87.8%)	34448(89.1%)	51176(88.9%)	41585(82.6%)
3-5	10969(10.8%)	3777(9.8%)	5756(10.0%)	7675(15.2%)
≥6	1354(1.3%)	443(1.1%)	624(1.1%)	1087(2.2%)

Table S2 | Corresponding patient characteristics among Internal and External Validation dataset.

	Internal validation (n = 57,556)	External validation (n = 50,347)	p-value
AGE	53.4±18.1	55.5±18.7	<0.001
GENDER (male)	28279(49.1%)	24563(48.8%)	0.257
CHA2DS2-VASc	1.7±1.6	2.1±1.9	<0.001
Diabetes mellitus	9150(15.9%)	11315(22.5%)	<0.001
End stage renal disease	1482(2.6%)	1345(2.7%)	0.322
Hypertension	16141(28.0%)	20656(41.0%)	<0.001
Coronary artery disease	8002(13.9%)	10227(20.3%)	<0.001
Peripheral arterial occlusion disease	900(1.6%)	1343(2.7%)	<0.001
Heart failure	2206(3.8%)	2766(5.5%)	<0.001
Transient ischemic attack	2169(3.8%)	2746(5.5%)	<0.001
Ischemic stroke	2864(5.0%)	3262(6.5%)	<0.001
Haemorrhagic stroke	1065(1.9%)	951(1.9%)	0.641
Chronic obstructive pulmonary disease	4517(7.8%)	7627(15.1%)	<0.001
Alcoholism	1024(1.8%)	829(1.6%)	0.094

Table S3 | The logistic regression coefficients of propensity score model.

	**Propensity score-1**<br>Case: with pre-existing AF (n = 98)<br>Control: without pre-existing AF (n = 13580)		**Propensity score-2**<br>Case: with postoperative NOAF within 1 month (n = 54)<br>Control: without postoperative NOAF within 1 month (n = 13526)	
	**Coefficient**	**Standard error**	**Coefficient**	**Standard error**
**Intercept**	-5.929	0.765	-6.305	0.974
**Surgery type**				
Low risk	Reference		Reference	
High risk	-0.465	0.271	-0.434	0.376
**Sex**				
Female	Reference		Reference	
Male	0.584	0.238	0.260	0.314
**Age (per 1 y/o)**	0.044	0.011	0.064	0.015
**CHA_2DS_2-VASc (per 1 score)**	0.101	0.094	-0.167	0.133
**RCRI (per 1 score)**	1.006	0.149	0.841	0.217

Table S4 | Baseline characteristics stratified by observed atrial fibrillation after inverse probability weighting.

	Pre-AF Group Pre-existing AF	NOAF Group new-onset AF within 30 days after operation	Control Group Other patients	SMD (Pre-AF vs. Control)	SMD (NOAF vs. Control)
**Surgery type (high risk)**	25.4%	28.7%	31.3%	-0.129	-0.056
**Hospital (community hospital)**	30.3%	17.8%	34.8%	-0.093	-0.357
**Sex (male)**	54.6%	52.7%	56.0%	-0.028	-0.067
**Age (y/o, mean±SD)**	64.9±14.4	64.4±18.5	67.0±15.6	-0.135	-0.163
**CHA_2DS_2-VASc (mean±SD)**	3.2±2.0	3.4±2.4	3.5±2.6	-0.086	-0.030
**CHA_2DS_2-VASc group**					
0	12.6%	3.5%	11.2%	0.045	-0.244
1	8.2%	26.8%	19.8%	-0.294	0.176
2	12.2%	14.2%	12.8%	-0.018	0.041
3	24.4%	13.6%	10.9%	0.435	0.089
4	20.1%	11.6%	10.2%	0.325	0.046
5	7.9%	7.8%	9.5%	-0.056	-0.057
6	8.3%	13.9%	9.6%	-0.044	0.146
7-9	6.3%	8.5%	16.0%	-0.263	-0.204
**RCRI (mean±SD)**	1.3±1.1	1.5±1.4	1.5±1.5	-0.121	-0.037
**RCRI group**					
0	20.9%	30.3%	36.8%	-0.330	-0.135
1	45.0%	32.1%	21.9%	0.557	0.246
2	19.7%	13.6%	13.4%	0.187	0.008
3	10.7%	9.8%	12.5%	-0.057	-0.082

	Pre-AF Group Pre-existing AF	NOAF Group new-onset AF within 30 days after operation	Control Group Other patients	SMD (Pre-AF vs. Control)	SMD (NOAF vs. Control)
4-5	3.7%	14.1%	15.3%	-0.325	-0.035
**Diabetes mellitus**	35.9%	42.1%	41.4%	-0.110	0.014
**Diabetes mellitus requiring insulin**	5.9%	10.6%	18.7%	-0.331	-0.208
**Serum creatinine ≥2 mg/dL**	33.4%	32.0%	30.8%	0.058	0.027
**End stage renal disease**	30.9%	26.4%	21.6%	0.228	0.117
**Hypertension**	66.1%	48.8%	55.2%	0.218	-0.129
**Coronary artery disease**	38.8%	33.9%	38.3%	0.010	-0.092
**Peripheral arterial occlusion disease**	14.5%	6.9%	9.7%	0.162	-0.094
**Heart failure**	29.8%	23.3%	17.3%	0.330	0.159
**Transient ischaemic attack**	3.9%	10.2%	14.9%	-0.310	-0.133
**Ischaemic stroke**	14.5%	14.1%	21.2%	-0.163	-0.174
**Haemorrhagic stroke**	16.1%	17.3%	7.2%	0.345	0.391
**Chronic obstructive pulmonary disease**	26.4%	13.6%	20.0%	0.159	-0.161
**Alcoholism**	6.3%	11.0%	3.0%	0.196	0.468

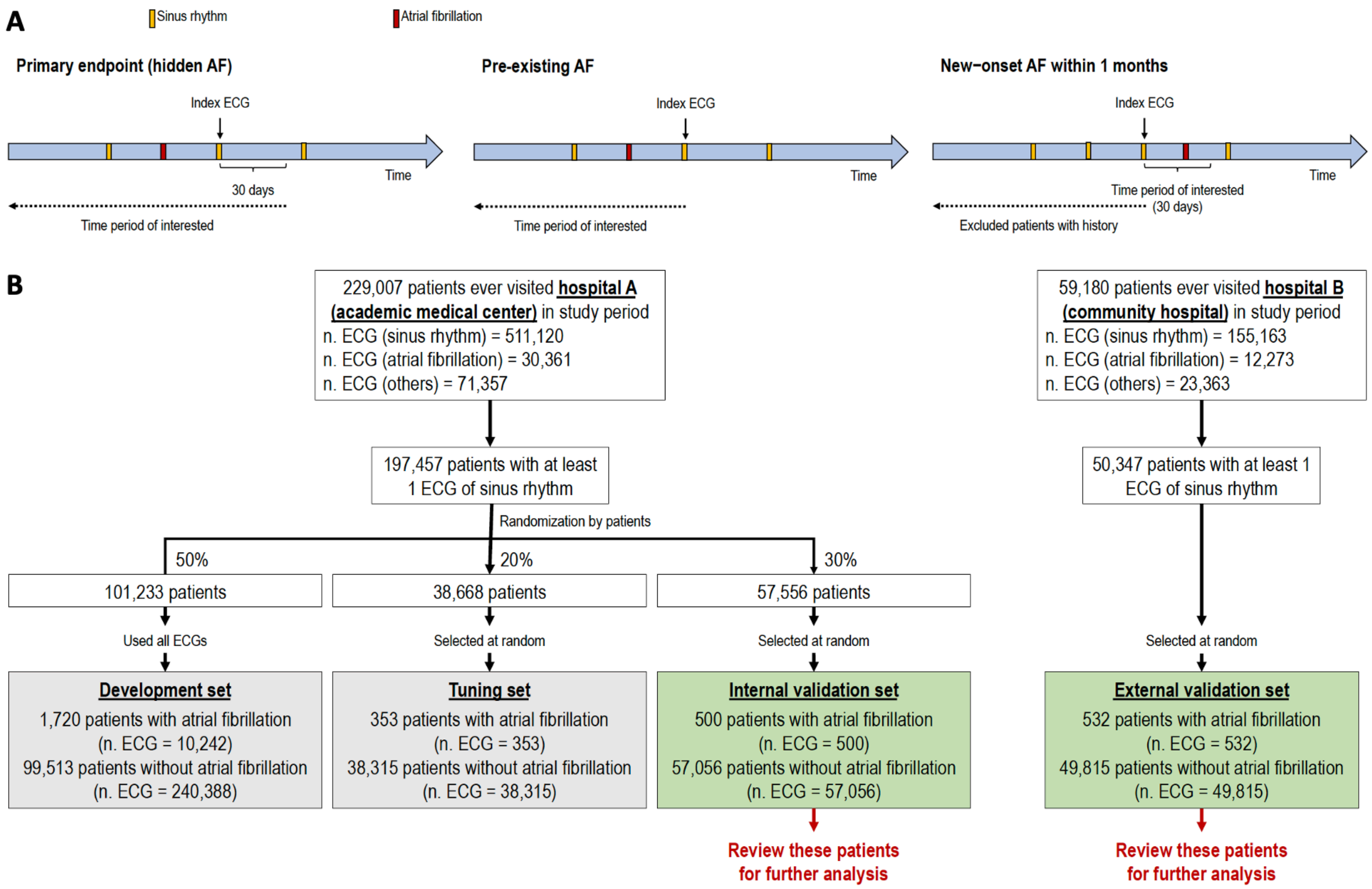

Figure S1 | Flow diagram for AI-ECG development. A) Windows of interest for patients with multiple ECGs. B) A schematic diagram of the dataset creation and analysis strategy devised to ensure a robust and reliable dataset for training, validating, and testing the network. Once the patient's data were placed in one of the datasets, the individual's data were used only in that set to avoid 'cross-contamination' among the development, training, and validation sets.

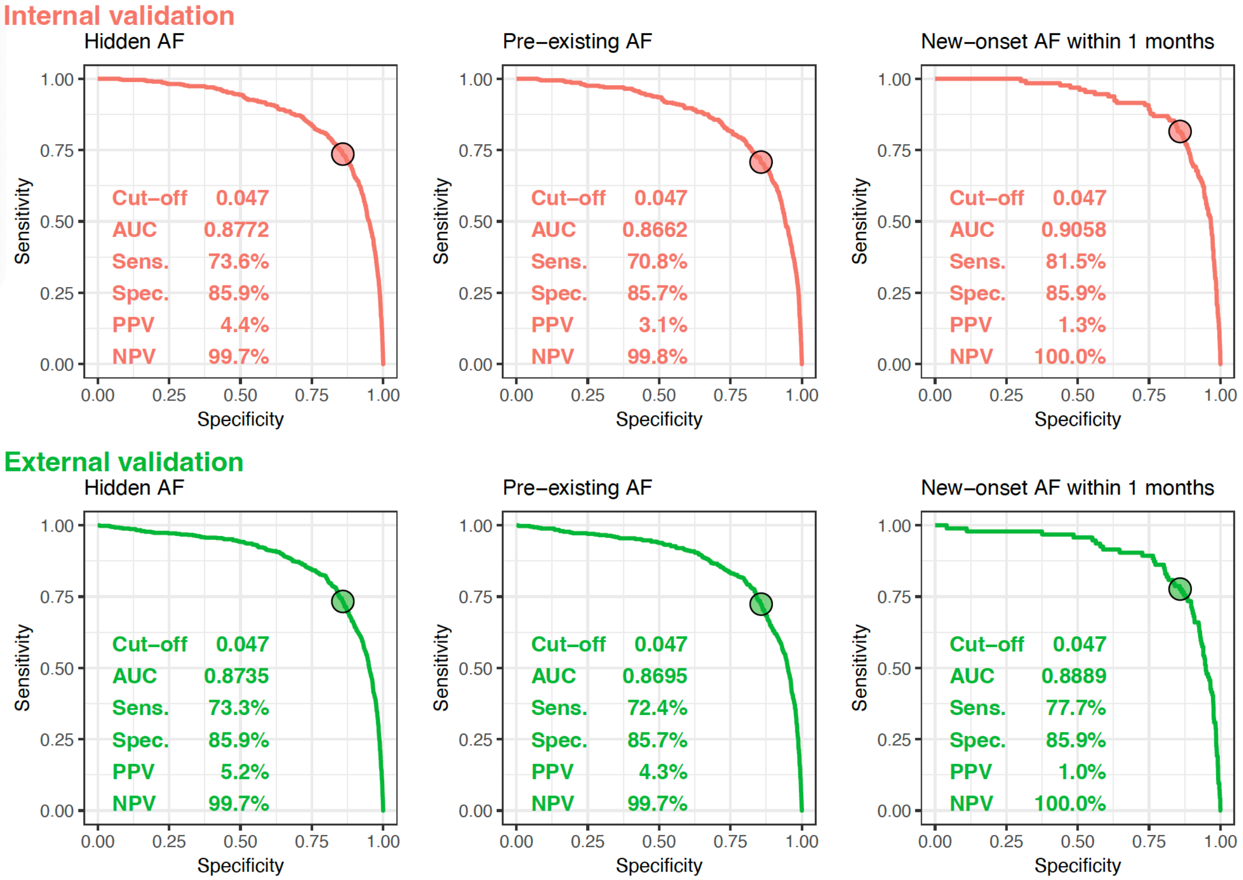

Figure S2 | The ROC curve of AI-ECG predictions to detect paroxysmal atrial fibrillation. The cut-off point was selected based on the maximum Youden's index in the training set and presented using a circle mark, which was defined as the threshold to distinguish between medium-to-high risk and low risk. Area under the ROC curve (AUC), sensitivity (Sens.), specificity (Spec.), positive predictive value (PPV), and negative predictive value (NPV) were calculated.

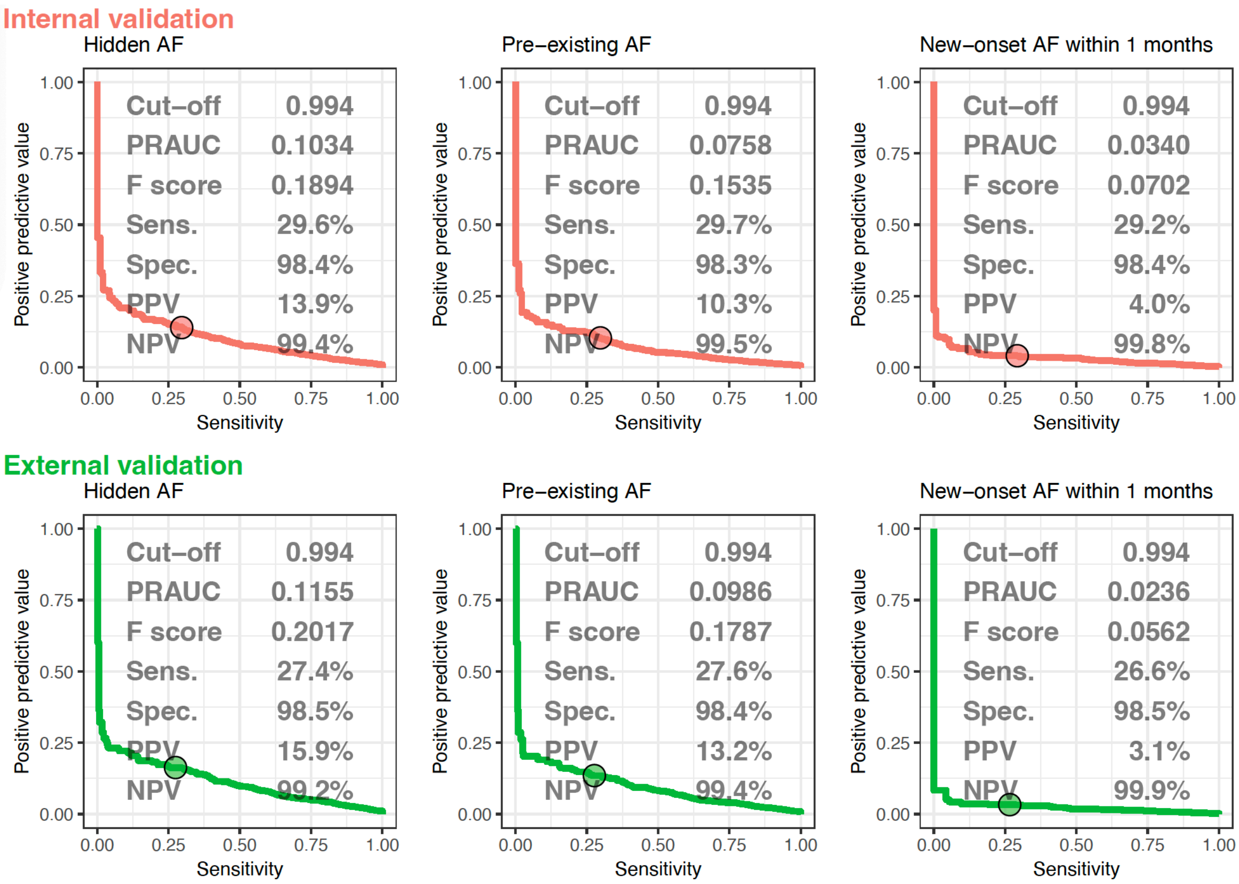

Figure S3 | The PRROC curve of AI-ECG predictions to detect paroxysmal atrial fibrillation. The cut-off point was selected based on the maximum F-score in the training set and presented using a circle mark, which was defined as the threshold to distinguish high risk from low to medium risk. Area under the ROC curve (AUC), sensitivity (Sens.), specificity (Spec.), positive predictive value (PPV), and negative predictive value (NPV) were calculated.

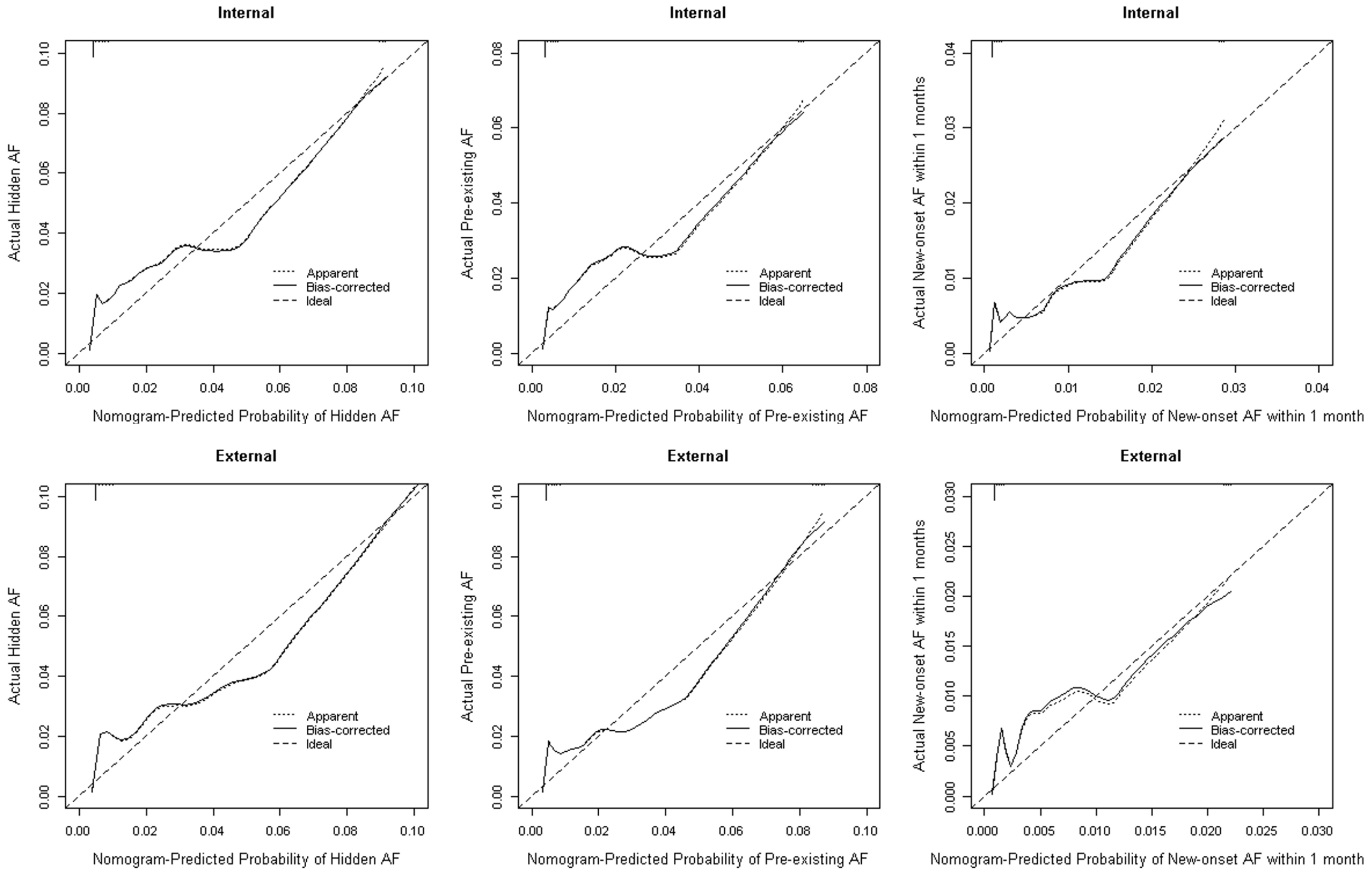

Figure S4 | Calibration Curves for AI-ECG Model Performance. These plots compare the predicted probabilities from the AI-ECG model with the actual observed event rates. The ideal line (dashed line) represents perfect calibration where predicted probabilities equal observed probabilities. The figures illustrate the agreement between predicted probabilities and observed event rates for the AI-ECG model in both internal and external validation cohorts across three AF categories: hidden AF, pre-existing AF, and new-onset AF within one month.

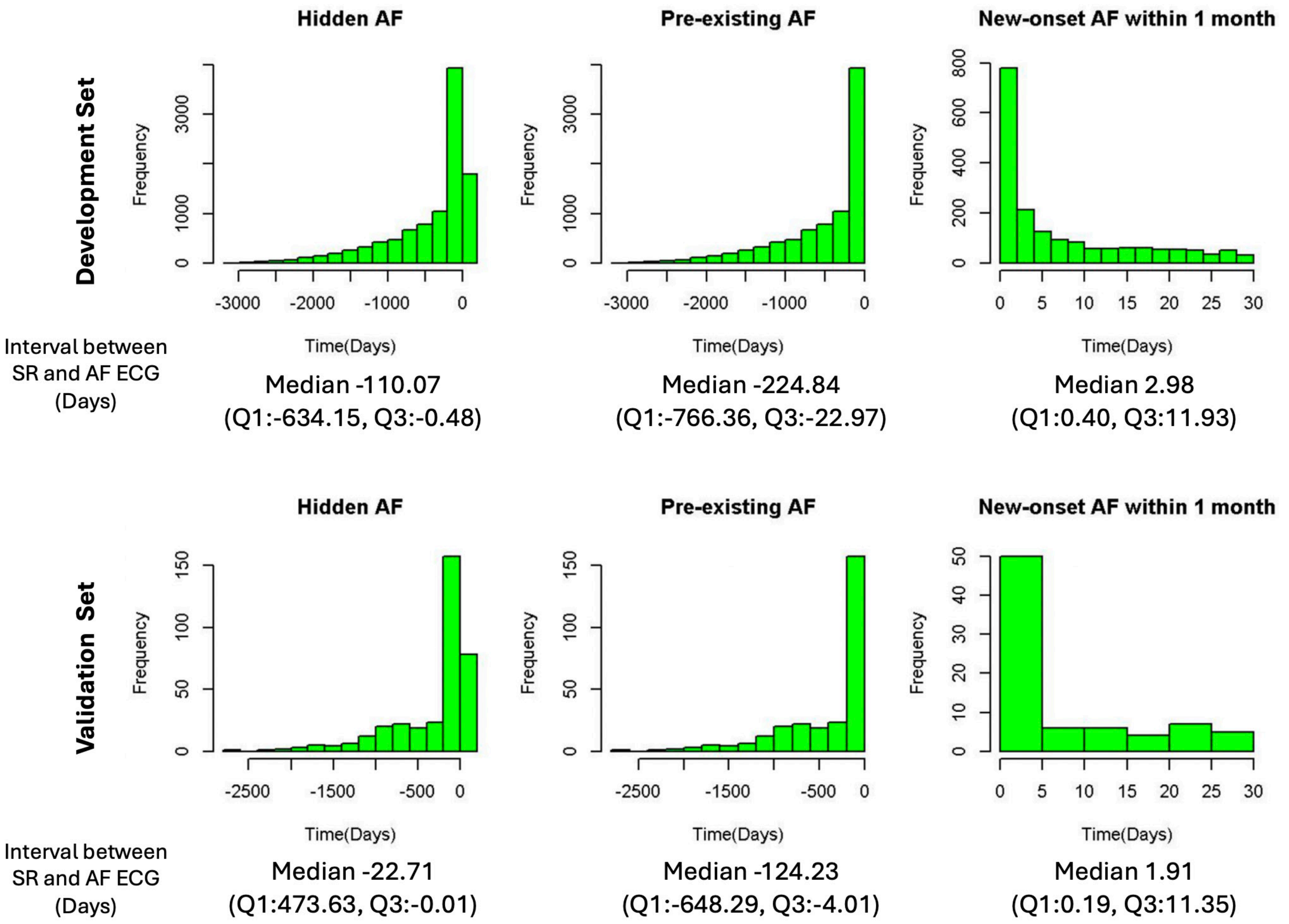

Figure S5 | The interval between sinus rhythm ECG and atrial fibrillation ECG.

Distribution of time intervals (in days) between sinus rhythm (SR) and atrial fibrillation (AF) ECGs in the development (top row) and validation (bottom row) cohorts. Negative values indicate AF ECGs recorded before SR ECGs. Median values and interquartile ranges (Q1–Q3) are shown for each subgroup.

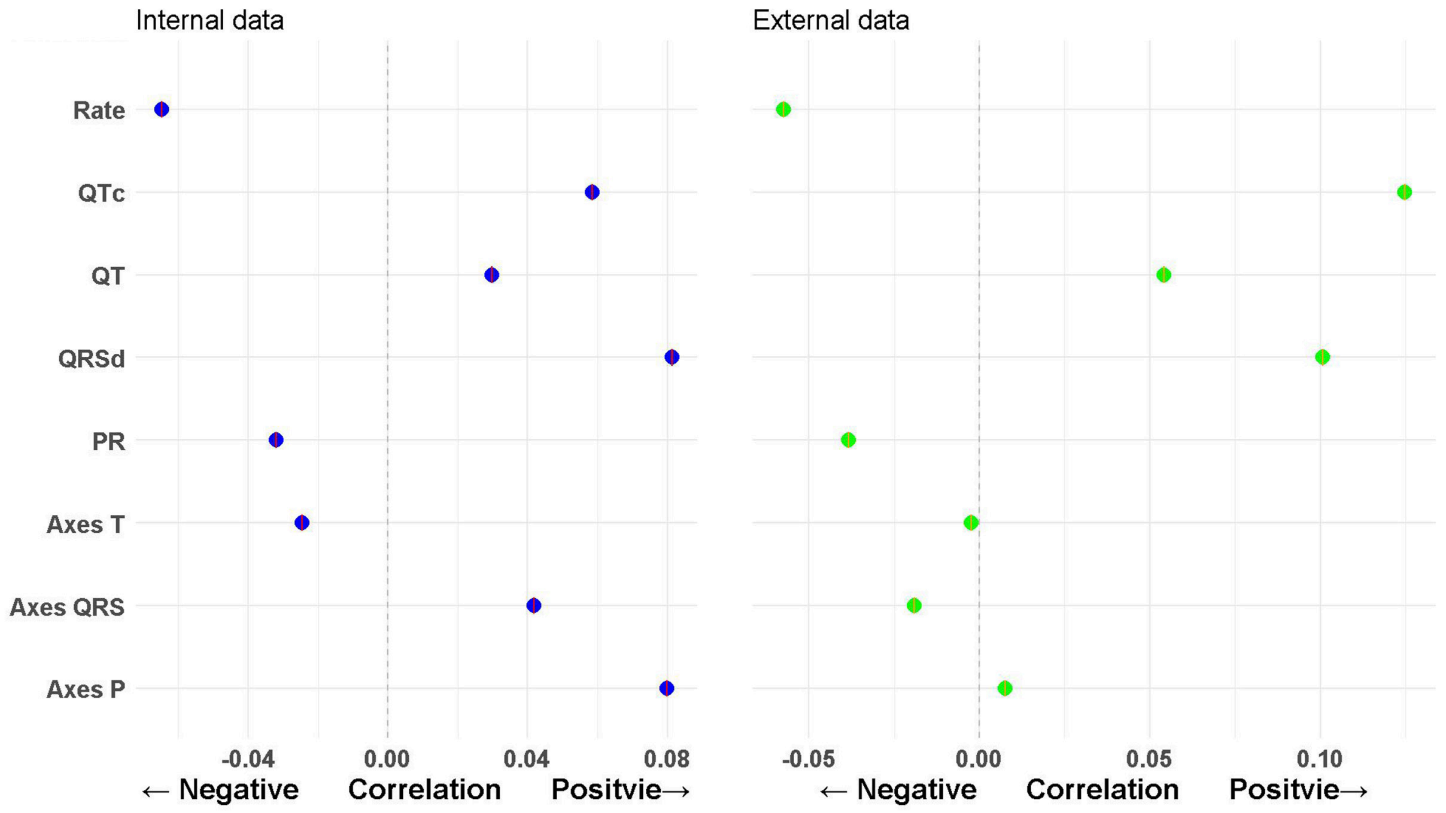

Figure S6 | Correlation of ECG Features with AI-ECG Model Predictions

The analysis illustrated the correlation coefficients between individual ECG features and the AI-ECG model's predictions in both the internal (blue dots, left panel) and external (green dots, right panel) validation datasets. Each point represents the strength and direction of correlation between a specific ECG parameter and the predicted AF risk score. Positive correlations mean higher feature values link to higher predicted risk, while negative correlations mean the opposite.

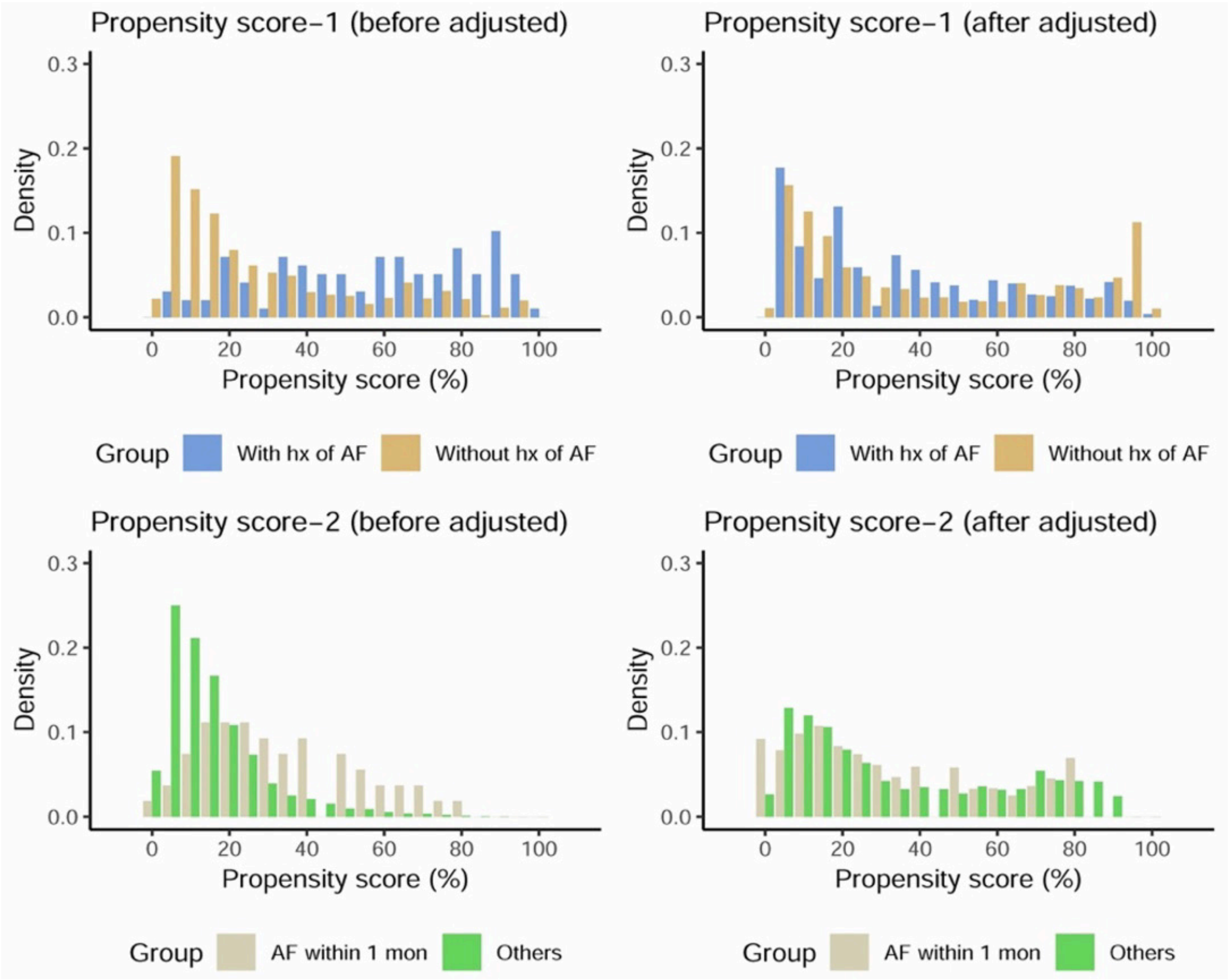

Figure S7 | The distribution of propensity score before and after inverse probability weighting. The density plots illustrate the propensity score distributions before and after weighting for both the pre-existing AF cohort (top row) and the postoperative NOAF cohort within 1 month (bottom row).Before inverse probability weighting, we observed that the distributions of propensity scores in the cases and controls were significantly different. Their distributions were significantly closer after inverse probability weighting, demonstrating the effect of propensity score processing on reducing the impact of confounding bias.

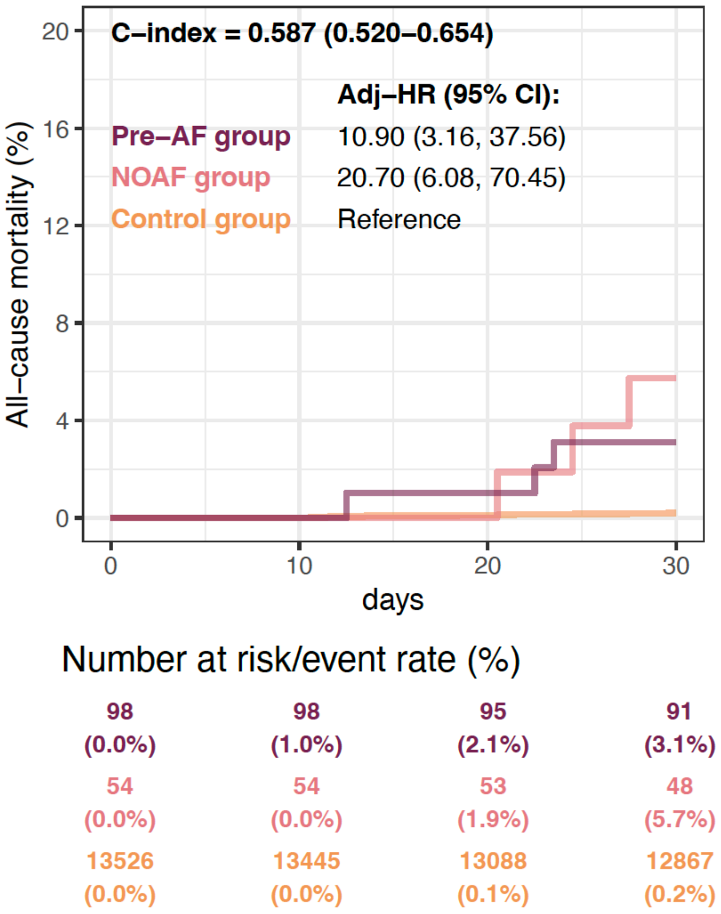

Figure S8 | The comparison between patients with and without observed atrial fibrillation adjusted by age and sex on all-cause mortality. This analysis included 13,678 patients to validate the results of the previous study.

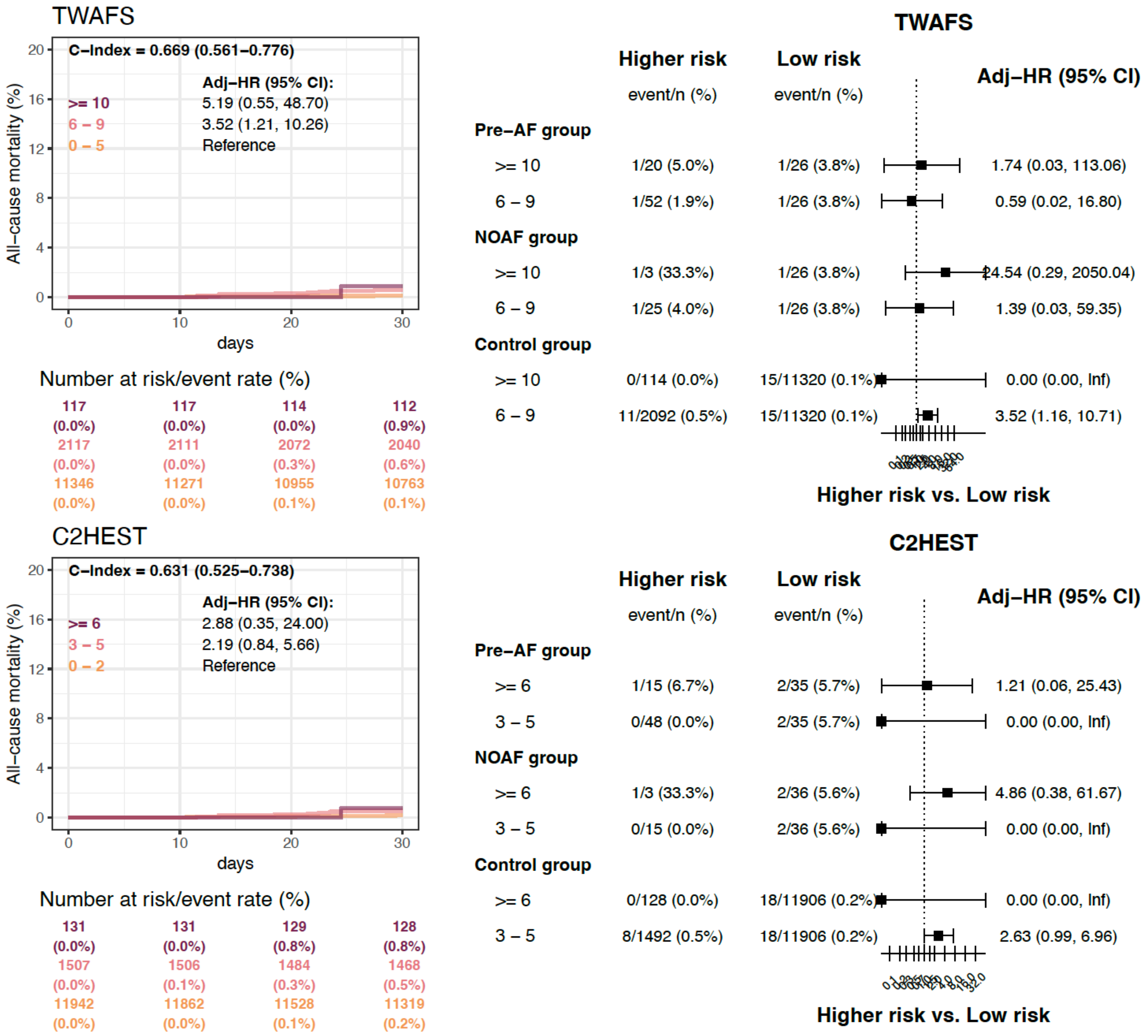

Figure S9 | The relationship between clinical scores and all-cause mortality. For the Kaplan–Meier curve analysis, we only included patients without a history of paroxysmal AF. The HRs were adjusted for age and sex.
